# Archaeal Community Structures in the Solfataric Acidic Hot Springs with Different Temperatures and Elemental Compositions

**DOI:** 10.1155/2013/723871

**Published:** 2013-04-22

**Authors:** Tomoko Satoh, Keiko Watanabe, Hideo Yamamoto, Shuichi Yamamoto, Norio Kurosawa

**Affiliations:** Division of Environmental Engineering for Symbiosis, Graduate School of Engineering, Soka University, 1-236 Tangi-machi, Hachioji, Tokyo 192-8577, Japan

## Abstract

Archaeal 16S rRNA gene compositions and environmental factors of four distinct solfataric acidic hot springs in Kirishima, Japan were compared. The four ponds were selected by differences of temperature and total dissolved elemental concentration as follows: (1) Pond-A: 93°C and 1679 mg L^−1^, (2) Pond-B: 66°C and 2248 mg L^−1^, (3) Pond-C: 88°C and 198 mg L^−1^, and (4) Pond-D: 67°C and 340 mg L^−1^. In total, 431 clones of 16S rRNA gene were classified into 26 phylotypes. In Pond-B, the archaeal diversity was the highest among the four, and the members of the order Sulfolobales were dominant. The Pond-D also showed relatively high diversity, and the most frequent group was uncultured thermoacidic spring clone group. In contrast to Pond-B and Pond-D, much less diverse archaeal clones were detected in Pond-A and Pond-C showing higher temperatures. However, dominant groups in these ponds were also different from each other. The members of the order Sulfolobales shared 89% of total clones in Pond-A, and the uncultured crenarchaeal groups shared 99% of total Pond-C clones. Therefore, species compositions and biodiversity were clearly different among the ponds showing different temperatures and dissolved elemental concentrations.

## 1. Introduction

The extreme environments are unique places to study how organisms interact with and adapt to the surroundings. Some of high temperature environments especially such as terrestrial hot springs and oceanic hydrothermal vents may resemble volcanic habitats that are thought to have existed on early Earth [1–3]. Indeed, some of the archaeal and bacterial lineages identified from hot springs appear to be related to lineages close to the root of the phylogenetic tree [4].

Hot spring microbial communities have been extensively studied in many areas such as Yellowstone National Park in the United States [5–10], Kamchatka hot springs in Russia [11], the island of the Lesser Antilles [12, 13], Icelandic hot springs [11, 14], Mt. Unzen hot springs in Japan [15], Ohwakudani hot springs in Japan [16], Pisciarelli hot springs in Italy [17], Bor Khlueng hot springs in Thailand [18], Wai-o-tapu geothermal area in New Zealand [19], and Tengchong hot springs in China [20]. These pioneering works enabled better appreciation of prokaryotic communities in the high temperature environments. However, despite decades of research, we still understand relatively little about the relationship between the environmental factors and hot spring prokaryotic community. It is important to reveal that which environmental factors affect prokaryotic community structures and diversity in individual hot spring habitats. Temperature has perhaps received the most attention, but other constraining factors may include pH, oxidation redox potential, elemental composition, and organic matter composition. In this study, we compared the archaeal community structures and diversity of four distinct solfataric acidic hot springs in Kirishima, Japan.

## 2. Materials and Methods

### 2.1. Sample Collection and Analyses of Dissolved Elemental Compositions

**Figure 1:**
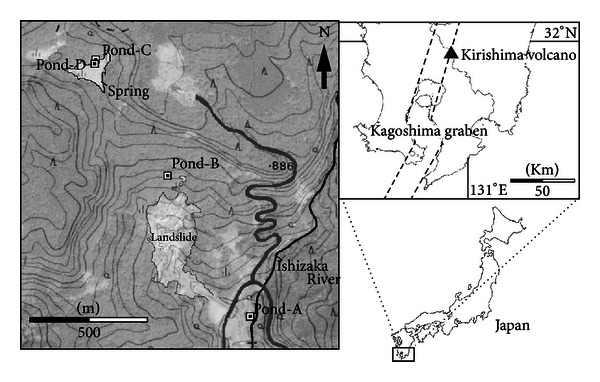
Map of sampling site in Kirishima geothermal area, Japan.

The investigated hot springs in this study are located in a field of one square kilometer in the Kirishima geothermal area in Japan (Kirishima National Park) (Figure 1, Table 1) where, the extensive volcanic activity occurred from the Pleistocene epoch to the present, depositing a thick pile of volcanic rocks [21]. Kirishima volcano, one of the largest Quaternary volcanoes in Japan, belongs to the northern part of the Kagoshima graben, a volcano-tectonic depression [22] caused by the subduction of the Philippine Sea plate. This volcano occupies an area of about 20 km × 30 km elongated in the northwest to southeast direction and contains more than 20 small volcanoes [23].

Sampling location within Kirishima geothermal area is a private land; therefore, people are usually not allowed to trespass on this area. We got permission to take hot spring water, soil, and various other native samples in this area from an owner of the land. There are many hot springs and muddy ponds showing a variety of temperatures and elemental compositions.

Muddy water sample was collected into sterile 100 mL glass bottle at each pond. Temperature and pH of the samples were measured at each sampling site. A part of each sample was filtered using 0.22 *μ*m membrane filter (Asahi Glass) and was subjected to analysis of dissolved elemental concentrations using the inductively coupled plasma optical emission spectroscopy (ICPS-7000 ver.2, Shimadzu). In the present study, we selected four ponds displaying a range of temperatures and dissolved elemental compositions for the archaeal community analysis. 

### 2.2. 16S rRNA Gene Clone Libraries and Sequencing

The environmental DNA was extracted from 5 to 10 g of each muddy water sample using the UltraClean Soil DNA Kit Mega Prep (Mo Bio Laboratories) according to the manufacturer's instructions. The precipitated DNA was purified using the GFX PCR DNA and Gel Band Purification Kit (GE Healthcare).

Purified DNA was used as the template for the amplification of archaeal 16S rRNA gene by archaea-specific primer A21F: 5′-TTCCGGTTGATCCYGCCGGA and universal primer U1492R: 5′-GGYTACCTTGTTACGACTT. The PCR conditions included an initial denaturation step at 94°C for 3 min, followed by 35 cycles of denaturation at 94°C for 30 sec, annealing at 55°C for 30 sec, and extension at 72°C for 2 min using Ex *Taq* DNA polymerase (Takara Bio). This was followed by a final extension at 72°C for 10 min.

The PCR products were purified using the aforementioned GFX Kit and were ligated into the pT7 Blue T-Vector (Novagen). *E. coli* DH5*α* cells were transformed with the plasmid library and were plated onto LB plates including 100 *μ*g mL^−1^ ampicillin, 40 *μ*g mL^−1^ X-gal, and 0.5 mM IPTG. Blue/white selection was conducted by randomly picking and subculturing individual white colonies in 100 *μ*L of 2 × YT medium containing 100 *μ*g mL^−1^ ampicillin in a 96-well plate at 37°C overnight. The inserted 16S rRNA gene was amplified using 1 *μ*L of the culture as the template with the same PCR procedure mentioned above. About 800 bp of the 5′-region of each 16S rRNA gene clone was sequenced by the aforementioned archaea-specific primer A21F and used for taxonomic and phylogenetic analysis.

### 2.3. Identification of 16S rRNA Gene Clones and Phylogenetic Analysis

16S rRNA gene sequences were edited using the MEGA5 (Molecular Evolutionary Genetics Analysis, http://www.megasoftware.net/) [24]. We also searched for chimera sequences by manually checking the sequence alignments using GENETYX ver. 10.0.3 software (Genetyx). Clones having 97% sequence similarity or higher were treated as a phylotype. The representative sequences of each phylotype were compared with 16S rRNA gene sequences published in the National Center for Biotechnology Information DNA database using BLAST (BLASTN; http://www.ncbi.nlm.nih.gov/BLAST/) [25] to identify individual clones. The representative sequences of each phylotype and related sequences in the GenBank data base were aligned using CLUSTALW ver. 1.83 program [26]. The maximum likelihood tree including bootstrap probabilities (1000 samplings) was constructed using the MEGA5. 

### 2.4. Statistical Analyses

Measurements of diversity ideally include richness, the number of different species or groups present, and evenness, the distribution of those groups [27, 28]. The Shannon-Weaver index [29], *H*′ = −Σ(*pi*)(ln⁡*pi*), and Simpson's reciprocal index [30], 1/*D*, where *D* = Σ(*pi*)^2^, and *pi* is the proportion of phylotypes *i* relative to the total number of phylotypes, both take richness and evenness into account [13, 28]. The Shannon-Weaver index and Simpson's reciprocal index were calculated using ESTIMATES 8.0 [31]. Evenness (*J*′ = *H*′/ln⁡*S*) was also calculated [32]. ESTIMATES 8.0 was also used to calculate Chao1 nonparametric richness estimator [33] and abundance-based coverage estimator of species richness (ACE) [34]. These coverage estimators determine the number of probable phylotypes in the environment compared with the numbers observed in the sample. The homologous coverage (biodiversity coverage) *C* was determined with the following equation: *C* = 1 − (*N*/*n*), where *N* is the number of phylotypes sequences and *n* is the total number of analyzed clones [35, 36]. Additionally, statistical analyses including principal components analysis to determine the correlations among the archaeal diversity with the environmental factors including temperature and dissolved elemental concentrations. Canonical correlation analysis was also performed to determine the correlations between archaeal groups and temperature or dissolved elemental concentrations, by using the software XLSTAT (Addinsoft, New York, NY). 

### 2.5. Nucleotide Sequence Accession Numbers

The representatives of nucleotide sequences of the phylotypes are available in the DDBJ/EMBL/GenBank databases under the accession numbers AB753272-AB753298 and AB755799-AB755806.

## 3. Results and Discussion

### 3.1. Water Chemistry

The four ponds in Kirishima geothermal area were selected based on the differences of temperatures and total dissolved elemental concentrations as follows: (1) Pond-A: 93°C and 1679 mg L^−1^, (2) Pond-B: 66°C and 2248 mg L^−1^, (3) Pond-C: 88°C and 198 mg L^−1^, and (4) Pond-D: 67°C and 340 mg L^−1^. The characteristics of sampling sites and these ponds are shown in Table 1. The range of pH values of the ponds was 2.0–2.6. In the ponds showing higher total dissolved elemental concentration, the concentrations and percentages of Fe, S, and Al were especially higher than those in other ponds. There was no significant difference of the concentrations of Mg, Si, Ca, P, Na, K, and as between the ponds.

### 3.2. 16S rRNA Gene Clone Libraries

16S rRNA gene clone libraries were successfully constructed using the environmental DNAs extracted from four muddy water samples. A total of 432 clones of archaeal 16S rRNA gene were analyzed. A chimerical sequence was detected during the analysis and was not used for further study. On the basis of the sequence similarity values, a total of 431 clones (Pond-A: 106, Pond-B: 112, Pond-C: 109, and Pond-D: 104 clones) were classified into 26 phylotypes, consisting of 25 crenarchaeal phylotypes and a single euryarchaeal one (Table 2). The homologous coverage values were 0.88 or above for all ponds indicating that approximately 90% of the 16S rRNA gene clones in these ponds could be considered in this study (Table 3). 

The guanine-plus-cytosine (G + C) content in the 16S rRNA gene sequences detected from 26 phylotypes in this study ranged from 56.6% to 69.0%, with an overall average of 62.4 ± 3.6%. According to Kimura et al., the growth temperature of archaea are strongly correlated with the G + C content, while the phylotypes containing this amount of G + C were grouped as the moderately thermophilic and hyperthermophilic archaea [37]. Therefore, all phylotypes detected in this study could possibly be related to moderately thermophilic and hyperthermophilic archaea.

### 3.3. Archaeal Community in Pond-A

On the basis of 16S rRNA gene sequence similarities, 106 clones derived from Pond-A which showed higher temperature and total dissolved elemental concentration consisted of five phylotypes of Crenarchaeota (Table 2). The 5% and 7% sequences of this pond were highly similar to those of cultured species (>98.0%) of the order Thermoproteales, *Caldivirga maquilingensis* and *Vulcanisaeta distributa*, respectively. The type strains of both species were hyperthermophilic archaea optimally growing at above 85°C, and they were originally isolated from acidic hot springs in Philippines and Japan, respectively [38, 39]. On the other hand, other 89% of Pond-A clones did not show significant similarities with any cultured species. Almost all the clones of them were assigned as a phylotype ST8A1-12 affiliated with the order Sulfolobales. This phylotype showed 95-96% sequence similarity with published environmental clones, NAKO74-07 and HS3wa_52 detected from Nakabusa hot spring, Japan (DNA database Accession no. AB366602) [37] and Tatung Volcano hot spring, Taiwan (DNA database Accession no. FJ797311). The diversity represented by the Shannon-Weaver index and Simpson's reciprocal index in the Pond-A was the lowest among the four ponds (Table 3).

### 3.4. Archaeal Community in Pond-B

**Figure 2:**
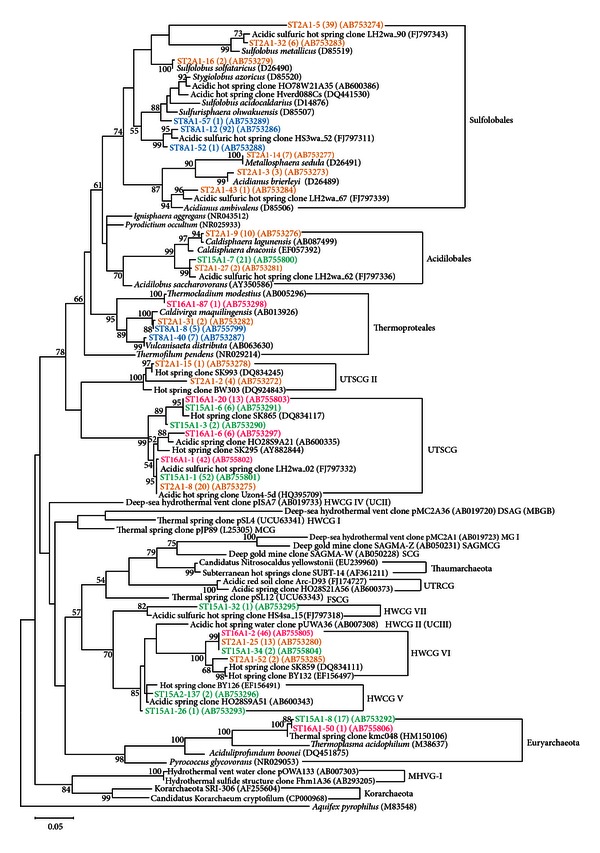
Phylogenetic tree of archaeal 16S rRNA gene sequences detected in Kirishima hot springs. Bootstrap values (>50%) based on 1000 replicates are indicated at nodes. The scale bar indicates the number of nucleotide substitutions per position. Number in the parenthesis with phylotype name represents the number of clones of each phylotype. The DNA database accession numbers are also indicated in the parenthesis. *Aquifex pyrophilus* is used as an outgroup species. The phylotype names derived from Pond-A, Pond-B, Pond-C, and Pond-D shown in blue, yellow, red, and green, respectively. UTSCG: uncultured thermoacidic spring clone group [16], HWCG I: hot water crenarchaeotic group I [6, 46], HWCG II (known as UCIII): uncultured crenarchaeal group III [15, 45, 46], HWCG IV (also known as UCII) [45, 49, 50], DSAG (known as MBGB): deep-sea archaeal group (marine benthic group B) [49, 51], MCG: miscellaneous crenarchaeal group [5, 50, 52, 53], MG I: marine crenarchaeotic group I [49, 54], SAGMCG: South Africa gold mine group [52], SCG: soil crenarchaeotic group [52], UTRCG: uncultured Thaumarchaeota-related clone group [16], FSCG: forest soil crenarchaeotic group [6, 55], and MHVG-I: marine hydrothermal vent group I [15, 16, 52].

In contrast to the Pond-A, the largest number of phylotypes was detected in Pond-B which was characterized as lower temperature and higher total dissolved elemental concentration, resulting, that the diversity indices and evenness value in this pond were highest among the four ponds (Table 3). A total of 112 clones consisted of 14 phylotypes that were classified into the following six groups: the order Sulfolobales, Acidilobales, Thermoproteales, and three uncultured crenarchaeal groups (Table 2, Figure 2). The 21% of the total clones were closely related to any of five cultured species (>98.0%): *Sulfolobus solfataricus* [40], *Metallosphaera sedula* [41], *Acidianus brierleyi *[42], *Caldisphaera lagunensis* [43], and *Caldivirga maquilingensis* [38]. *S. solfataricus*, *M*.* sedula,* and *A*.* brierleyi* are facultatively chemolithoautotrophic aerobes and require elemental sulfur or sulfidic ores. These species and their close relatives have been isolated from acidic Solfatara fields around the world [44]. *C*.* lagunensis* and *C*.* maquilingensis* are heterotrophic anaerobes. Their growths are stimulated or constrained by the presence of sulfur as an electron acceptor.

On the other hand, nine phylotypes sharing 79% in total of all Pond-B clones showed no significant similarity with any cultured species. Nearly half of these uncultured clones were assigned as a phylotype ST2A1-5. This phylotype was most dominant (35%) in Pond-B and was phylogenetically distant not only from any cultured species but also from any published environmental clones. This novel phylotype belonged to a cluster in the order Sulfolobales (Figure 2). This cluster also harbored another Pond-B phylotype ST2A1-32, which showed 98% 16S rRNA gene sequence similarity with published environmental clone, LH2wa_90 detected from Taiwanese hot spring (DNA database Acc. no. FJ797343).

The phylotype ST2A1-8 belonging to the uncultured thermoacidic spring clone group (UTSCG) [16] was secondary dominant in Pond-B and it shared 18% of the total Pond-B clones. Interestingly, phylotypes similar to ST2A1-8 were also frequently detected in Pond-C and Pond-D, suggesting that this crenarchaeal species survive relatively wide range of temperature and dissolved elemental composition in acidic hot springs. There might be unfavorable factors in Pond-A for the presence of UTSCG. The phylotypes ST2A1-2 and ST2A1-15 were placed in the sister cluster of UTSCG with published clones detected from Yellowstone National Park (DNA database Acc. no. DQ834245). We call this cluster as UTSCG II in this study.

The phylotype ST2A1-25 was thirdly dominant in Pond-B and was placed into the sister cluster of the hot water crenarchaeotic group II (HWCG II) [15, 45, 46] with phylotype ST2A1-52 and published environmental clone SK859 detected from acidic hot spring in Yellowstone National Park (DNA database Acc. No. DQ834111). We call this cluster as HWCG VI in this study. The phylotype ST2A1-25 was also dominant in Pond-C.

### 3.5. Archaeal Community in Pond-C

The other high temperature pond, Pond-C, showed relatively low value of species richness as same as Pond-A (Table 3). A hundred and nine clones were classified into six phylotypes as follows: *Thermocladium modestius* of the order Thermoproteales, four uncultured crenarchaeal phylotypes and uncultured euryarchaeal phylotypes. The type strain of *T*. *modestius* was originally isolated from solfataric mud at Noji-onsen, Japan, and is an anaerobic heterotroph growing optimally around 75°C, pH 4.0 [47].

As mentioned in the previous section, the uncultured phylotypes ST2A1-8 of UTSCG and ST2A1-25 of HWCG VI were dominant in the clone library constructed for this pond sample. These two phylotypes shared 81% in total of the Pond-C clones. Three phylotypes sharing 56% of Pond-C clones were affiliated with uncultured thermoacidic spring clone group (UTSCG) [16].

### 3.6. Archaeal Community in Pond-D

A hundred and four clones derived from Pond-D which showed lower temperature and total dissolved elemental concentration consisted of nine phylotypes. The diversity indices in Pond-D were lower than those in Pond-B but were higher than the values in Pond-A and Pond-C (Table 3). The phylotype sharing 20% of the total clones was related to *Caldisphaera draconis* with 95% sequence similarity of 16S rRNA gene. This species is chemoorganotrophic anaerobe isolated from acidic hot spring in Yellowstone National Park [48].

Other phylotypes showed no significant similarity with any cultured species. The most frequent phylotype was ST2A1-8 affiliated with UTSCG and it shared 50% of the total clone in this pond. In contrast to the archaeal communities in other three ponds, the secondary dominant uncultured phylotype (ST16A1-50) was affiliated with Euryarchaeota and showed 99% sequence similarity of 16S rRNA gene with thermal spring clone kmc048 detected from Kamchatka hot springs in Russia (DNA database Acc. no. HM150106). This phylotype shared 16% of the total clones in this pond. 

The phylotype ST15A1-26 together with ST15A2-137 and ST15A1-32 were barely detected in Pond-D, and were placed into the sister cluster of HWCG II. We call these clusters as HWCG V and HWCG VII, respectively, in this study.

### 3.7. Archaeal Diversity and Community Structures with Different Temperatures and Total Dissolved Elemental Concentrations

When the diversity was compared within ponds with different temperatures (Temperature approximately 90°C, Pond-A + Pond-C versus Temp. approx. 70°C, Pond-B + Pond-D), represented by the Shannon-Weaver index and Simpson's reciprocal index, the lower temperature ponds showed higher diversity (Table 3). On the other hand, when comparing within ponds with different total dissolved elemental concentrations, the diversity indices of higher total dissolved elemental concentration ponds (El. conc. > 1600 mg L^−1^, Pond-A + Pond-B), were higher than those of Pond-C + Pond-D (El. conc. < 350 mg L^−1^). As a result, the archaeal diversity was the highest in the pond characterized as lower temperature and higher total dissolved elemental concentration (Pond-B). In contrast, the combination of higher temperature and lower total dissolved elemental concentration (Pond-A) caused the lowest diversity in this study. 

When focusing on the species composition and distribution, they were dissimilarity within ponds with different temperatures and total dissolved elemental concentrations. As shown in Table 2, the phylotypes affiliated with the order Sulfolobales were only detected in the ponds showing higher total dissolved elemental concentrations (Pond-A + Pond-B). The members of the order Sulfolobales are generally characterized as facultatively or obligately chemolithoautotrophic S^0^ metabolizers; some members oxidize ferrous iron and sulfidic ores, producing soluble metal sulfates [44]. Therefore, the presence of Sulfolobales makes sense in these ponds including higher total dissolved elemental concentration, especially sulfur, as we would expect to detect microbes that metabolically depend on sulfur as an electron donor. It is also interesting that the species compositions within the order Sulfolobales between Pond-A and Pond-B were clearly different from each other. We detected sequences closely related to Sulfolobales species (98.9% similarity) in Pond-B (66°C), but most of the clones detected from Pond-A (93°C) were affiliated with uncultured Sulfolobales, forming a phylotype ST8A1-12. In addition, the members of the genus *Caldisphaera* of the order Acidilobales were frequently detected from lower temperature ponds (Pond-B + Pond-D) but not detected from higher temperature ponds (Pond-A + Pond-C). This may be due to the growth temperature limit of the members of the genus *Caldisphaera*, which is 85°C [56]. 

### 3.8. Geochemistry and Archaeal Diversity or Groups Correlations

**Figure 3:**
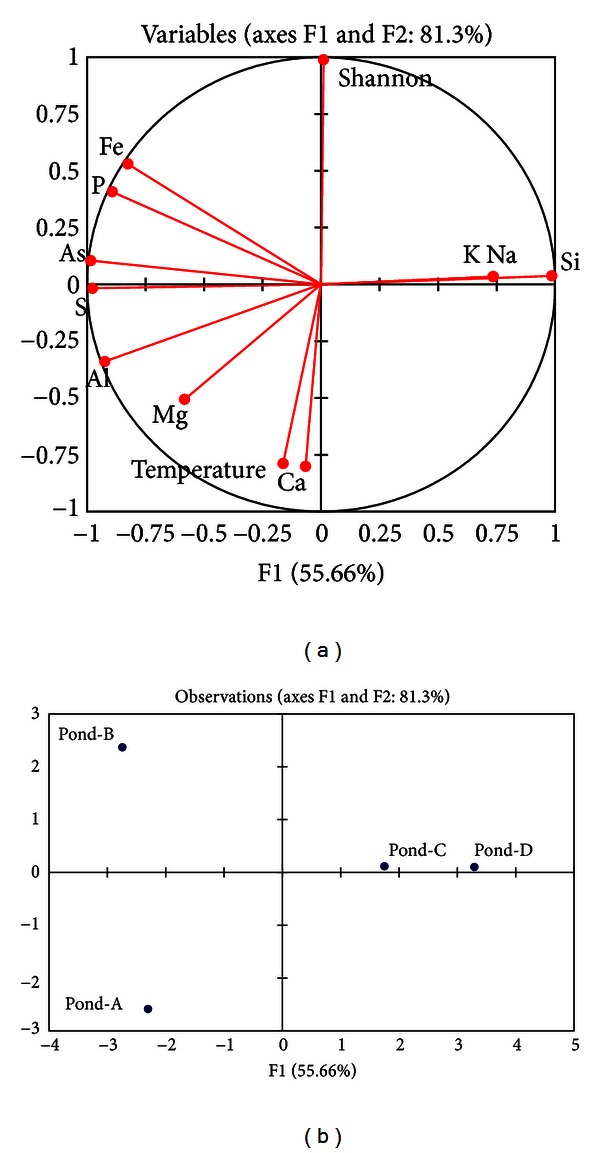
Principal components analysis showing loadings on principal components 1 and 2 for environmental factors at sites and the relationship to each site. Shannon, Temp. and El. Conc. indicate Shannon-Weaver index, temperature, and total dissolved elemental concentration, respectively.

A matrix was created based on Pearson's correlation coefficients (*r*) calculated from the Shannon-Weaver index, temperatures, and dissolved elemental concentrations at these four ponds (Table 4). Several dissolved elemental concentrations were statistically correlated with each other, but the archaeal diversity did not correlate with temperature and any dissolved elemental concentrations (significance level, *α* = 0.10). Total dissolved elemental concentration was strongly correlated with S, P, and As (*P* value < 0.05) and moderately correlated with Fe (*P* < 0.10). Al and S were moderately correlated with each other. Principal components analysis showed that axes F1 and F2 accounted for 81.3% of the variation between sites; S, As, and Si contributed equally to PC1, while the archaeal diversity index contributed to PC2 (Figure 3). Pond-A and Pond-B were higher in total dissolved elemental concentration, and variations at Pond-A and Pond-B appeared to be explained best by S and As of PC1, whereas the variations at Pond-A with higher temperature were best explained by PC2. 

**Figure 4:**
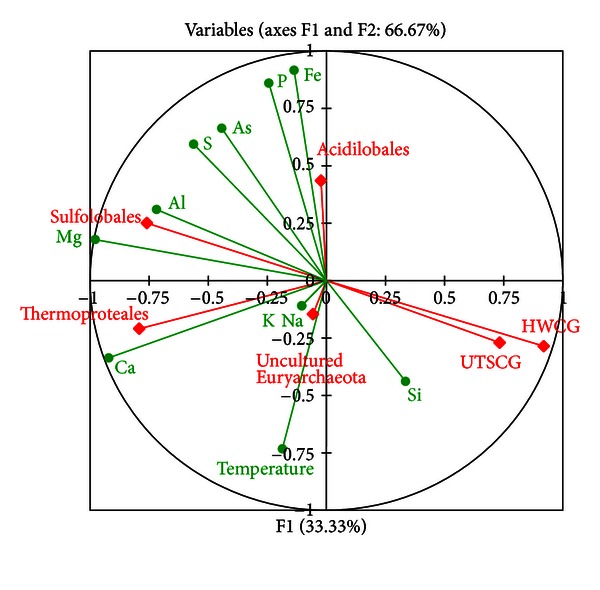
Canonical correlation analysis showing correlative relationships between environmental factors and proportions of individual archaeal groups. Archaeal groups were shown in abbreviations and rhombus. Environmental factors were shown in circle.

Canonical correlation analysis showed that some archaeal groups strongly correlate with particular environmental factors, such as the Sulfolobales with Al and uncultured Euryarchaeota with Na and K, whereas UTSCG group was negatively correlated with Al (*P* < 0.05) (Figure 4). The order Sulfolobales was also moderately correlated with S (*P* < 0.10). Moreover, the order Acidilobales, UTSCG, and HWCG groups were moderately negatively correlated with temperature, S, and Mg, respectively. To date, the element requirements in archaea were conducted on certain cultured species and little was known of the uncultured archaea. However, the correlations between the uncultured archaeal groups and the dissolved elemental concentrations shown in this study could give more insights into how specific elements affect uncultured archaeal communities.

## 4. Conclusion

In this study, we have investigated the archaeal community structures of four distinct solfataric acidic hot springs in Kirishima, Japan. The species compositions and biodiversity were clearly different among the ponds showing different temperatures and dissolved elemental concentrations. Although other environmental factors also could have influenced on the archaeal community structures, the present study will be helpful in understanding the archaeal ecology in the solfataric acidic hot springs.

## Figures and Tables

**Table 1:** Characteristics of sampling sites and ponds.

	Pond-A		Pond-B		Pond-C		Pond-D	
Temp (°C)	93		66		88		67	
pH	2.6		2.0		2.4		2.3	
Concentration (mg L^−1^)/composition (%)								
Fe	388.9	23	1149	51	9.630	5	27.18	8
S	663.2	40	702.8	31	59.76	30	61.90	18
Al	433.6	26	287.9	13	14.57	7	2.021	1
Mg	86.74	5	46.77	2	0.001	0	43.35	13
Si	47.88	3	45.52	2	103.9	53	148.4	44
Ca	54.81	3	10.88	0	7.498	4	39.26	12
P	2.850	0	4.711	0	1.265	1	1.266	0
Na	0.001	0	0.001	0	0.001	0	8.442	2
K	0.001	0	0.001	0	0.001	0	7.384	2
As	1.079	0	1.137	0	0.879	0	0.856	0
Total	**1679**	**100**	**2248**	**100**	**197.5**	**100**	**340.1**	**100**
Latitude (N)	31°54′37.7′′		31°54′52.4′′		31°55′05.0′′		31°55′04.5′′	
Longitude (E)	130°49′00.6′′		130°48′50.3′′		130°48′41.1′′		130°48′41.0′′	
Altitude (m)	759		842		884		885	
Color of sediments	Light brown		Light brown		Gray		Gray	

Detection limit is 0.001 mg L^−1^.

**Table 2:** Affiliation and closest published species or clones of 26 phylotypes.

Phylotypes	Affiliation	Closest species or clones (accession number)	16S rRNA gene similarity	Number of clones detected from each site			
			(%)	Pond-A	Pond-B	Pond-C	Pond-D
Order Sulfolobales							

ST2A1-3	*Acidianus brierleyi *	*Acidianus brierleyi* (D26489)	98.9		3		
ST2A1-43	*Acidianus* sp.	*Acidianus ambivalens* (D85506)	95.5		1		
ST2A1-14	*Metallosphaera sedula *	*Metallosphaera sedula* (D26491)	100.0		7		
ST2A1-16	*Sulfolobus solfataricus *	*Sulfolobus solfataricus* (D26490)	99.1		2		
ST8A1-57	*Sulfurisphaera* sp.	*Sulfurisphaera ohwakuensis* (D85507)	95.1	1			
ST2A1-5	Uncultured Sulfolobales	Acidic hot spring clone HO78W21A35 (AB600386)	80.7		39		
ST2A1-32	Uncultured Sulfolobales	Acidic sulfuric hot spring clone LH2wa_90 (FJ797343)	98.3		6		
ST8A1-12	Uncultured Sulfolobales	Acidic sulfuric hot spring clone HS3wa_52 (FJ797311)	96.6	92			
ST8A1-52	Uncultured Sulfolobales	Acidic sulfuric hot spring clone HS3wa_52 (FJ797311)	94.1	1			

Order Acidilobales							

ST2A1-9	*Caldisphaera lagunensis *	*Caldisphaera lagunensis* (AB087499)	98.5		10		
ST2A1-27	*Caldisphaera* sp.	*Caldisphaera draconis* (EF057392)	95.4		2		21
(=ST15A1-7)							

Order Thermoproteales							

ST8A1-8	*Caldivirga maquilingensis *	*Caldivirga maquilingensis* (AB013926)	98.0	5	2		
(=ST2A1-31)							
ST8A1-40	*Vulcanisaeta distributa *	*Vulcanisaeta distributa* (AB063630)	98.9	7			
ST16A1-87	*Thermocladium modestius *	*Thermocladium modestius* (AB005296)	99.4			1	

Other crenarchaeal groups							

ST2A1-8	UTSCG	Acidic sulfuric hot spring clone LH2wa_02 (FJ797332)	99.7		20	42	52
(=ST16A1-1, ST15A1-1)							
ST15A1-3	UTSCG	Acidic hot spring clone Uzon4-5d (HQ395709)	96.1				2
ST16A1-6	UTSCG	Acidic spring clone HO28S9A21 (AB600335)	96.9			6	
ST16A1-20	UTSCG	Hot spring clone SK865 (DQ834117)	96.9			13	6
(=ST15A1-6)							
ST2A1-2	UTSCG II	Hot spring clone BW303 (DQ924843)	93.3		4		
ST2A1-15	UTSCG II	Hot spring clone SK993 (DQ834245)	99.7		1		
ST15A1-26	HWCG V	Acidic spring clone HO28S9A51 (AB600343)	96.3				1
ST15A2-137	HWCG V	Acidic spring clone HO28S9A51 (AB600343)	98.4				2
ST2A1-25	HWCG VI	Hot spring clone SK859 (DQ834111)	95.8		13	46	2
(=ST16A1-2, ST15A1-34)							
ST2A1-52	HWCG VI	Hot spring clone SK859 (DQ834111)	96.0		2		
ST15A1-32	HWCG VII	Acidic sulfuric hot spring clone HS4sa_15 (FJ797318)	91.2				1

Euryarchaeal groups							

ST16A1-50	Uncultured Euryarchaeota	Thermal spring clone kmc048 (HM150106)	99.2			1	17
(=ST15A1-8)							

Total				106	112	109	104

**Table 3:** Diversity index scores for clone libraries.

Sample	Shannon	Simpson	Rich	Even	*S*_ACE_	*S*_Chao1_	Coverage	Total clone number
Pond-A	0.53	1.32	5	0.332	7.04	6.00	0.95	106
Pond-B	2.06	5.41	14	0.780	15.4	14.2	0.88	112
Pond-C	1.23	2.91	6	0.687	10.1	7.00	0.94	109
Pond-D	1.45	3.10	9	0.659	10.9	9.25	0.91	104
Temperature approximately 90°C (Pond-A + Pond-C)	1.58	3.66	11	0.659	16.0	17.0	0.95	215
Temperature approximately 70°C (Pond-B + Pond-D)	2.20	5.82	20	0.735	23.3	21.0	0.91	216
El. conc. > 1600 ppm (Pond-A + Pond-B)	1.99	4.37	18	0.689	21.0	19.5	0.92	218
El. conc. < 350 ppm (Pond-C + Pond-D)	1.61	3.68	11	0.673	15.5	13.3	0.95	213

Diversity index scores measured were Shannon-Weaver index (Shannon), Simpson's reciprocal index (Simpson), Richness (Rich), Evenness (Even), the coverage estimators *S*_ACE_ and *S*_Chao1_, and the homologous coverage. El. conc. indicates total dissolved elemental concentration.

**Table 4:** Correlation matrix showing *r* values for Pearson's correlation.

Variables	Shannon	Temp.	Fe	S	Al	Mg	Si	Ca	P	Na	K	As	El. Conc.
Shannon	**1.00 **	−0.87	0.54	0.00	−0.32	−0.41	0.07	−0.71	0.42	0.14	0.14	0.11	0.20
Temp.	−0.87	**1.00 **	−0.40	0.05	0.31	0.12	−0.29	0.29	−0.29	−0.55	−0.55	−0.01	−0.14
Fe	0.54	−0.40	**1.00 **	0.84	0.62	0.34	−0.76	−0.26	**0.99 **	−0.46	−0.46	**0.90 **	**0.92 **
S	0.00	0.05	0.84	**1.00 **	**0.95 **	0.71	−**0.93 **	0.21	**0.91 **	−0.57	−0.57	**0.99 **	**0.98 **
Al	−0.32	0.31	0.62	**0.95 **	**1.00 **	0.82	−**0.90 **	0.44	0.72	−0.57	−0.57	**0.90 **	0.87
Mg	−0.41	0.12	0.34	0.71	0.82	**1.00 **	−0.49	0.82	0.44	−0.02	−0.02	0.61	0.64
Si	0.07	−0.29	−0.76	−**0.93 **	−**0.90 **	−0.49	**1.00 **	0.00	−0.83	0.84	0.84	−**0.94 **	−0.89
Ca	−0.71	0.29	−0.26	0.21	0.44	0.82	0.00	**1.00 **	−0.15	0.33	0.33	0.07	0.08
P	0.42	−0.29	**0.99 **	**0.91 **	0.72	0.44	−0.83	−0.15	**1.00 **	−0.51	−0.51	**0.95 **	**0.97 **
Na	0.14	−0.55	−0.46	−0.57	−0.57	−0.02	0.84	0.33	−0.51	**1.00 **	**1.00 **	−0.62	−0.51
K	0.14	−0.55	−0.46	−0.57	−0.57	−0.02	0.84	0.33	−0.51	**1.00 **	**1.00 **	−0.62	−0.51
As	0.11	−0.01	**0.90 **	**0.99 **	**0.90 **	0.61	−**0.94 **	0.07	**0.95 **	−0.62	−0.62	**1.00 **	**0.99 **
El. conc.	0.20	−0.14	**0.92 **	**0.98 **	0.87	0.64	−0.89	0.08	**0.97 **	−0.51	−0.51	**0.99 **	**1.00 **

Values in bold are different from 0 with a significance level **α** = 0.10. Diversity indices showed very similar correlations with each other and environmental variables: only Shannon-Weaver index for archaeal clone libraries is shown. Shannon, Temp. and El. Conc. indicate Shannon-Weaver index, Temperature, and total dissolved elemental concentration, respectively.
